# Clinical efficacy of high-flow nasal oxygen in patients undergoing ERCP under sedation

**DOI:** 10.1038/s41598-020-79798-7

**Published:** 2021-01-11

**Authors:** Boram Cha, Man-Jong Lee, Jin-Seok Park, Seok Jeong, Don Haeng Lee, Tae Gyu Park

**Affiliations:** 1 Division of Gastroenterology, Department of Internal Medicine, Inha University School of Medicine, 27 Inhang-ro, Jung-gu, Incheon, 400-711 Republic of Korea; 2 Division of Critical Care Medicine, Department of Hospital Medicine, Inha University School of Medicine, Incheon, Republic of Korea

**Keywords:** Biological techniques, Medical research

## Abstract

Hypoxemia can occur during endoscopic retrograde cholangiography (ERCP) and it is difficult to achieve adequate ventilation with the prone position. High-flow nasal oxygen (HFNO) has been recommended to be more effectively help ventilation than conventional low flow oxygen. The aim of this study was to evaluate the effect of HFNO during sedated ERCP and to identify predictors of desaturation during ERCP. The investigated variables were age, gender, American Society of Anesthesiologists classes (ASA), duration of exam, and sedative used for midazolam or/and propofol of 262 patients with sedated ERCP. The differences between categorical and continuous variables were analyzed using the Student’s *t* test and the *chi-*square test. Desaturation (SpO_2_ ≤ 90%) occurred in 9(3.4%) patients among 262 patients during sedated ERCP. The variables found to predict desaturation were older age (*p* < 0.01), higher sedation dose for midazolam or propofol (*p* < 0.01), and use of midazolam (*p* < 0.01). Desaturation rate was lower during sedated ERCP with HFNO compared to the preliminary study with conventional low flow nasal oxygen. Patients with older age, higher sedation dose, or the use of midazolam might require close monitoring for desaturation and hypoventilation by nursing staff. The study shows the use of high-flow nasal oxygen reduces the incidence of desaturation during ERCP.

## Introduction

Sedation and analgesia are critical components of gastrointestinal (GI) endoscopy, as patients often experience anxiety, pain, or discomfort. Endoscopic retrograde cholangiography (ERCP) is associated with a high frequency of procedure-associated complications, such as arterial hypoxemia, arrhythmias, and myocardial ischemia^1–4^, and the need to minimize the number of diagnostic procedures has been emphasized^5–7^.

Hypoxemia is the most common adverse cardiopulmonary complication during sedated endoscopy and is caused by respiratory depression, airway obstruction, and decreased chest wall compliance^8^. Although sedative agents, such as Midazolam and/or propofol are commonly used for sedative endoscopy in clinical practice, respiratory depression is frequently encountered because of blunting central chemoreceptor responsiveness to CO_2_^9^_,_ and alveolar hypoventilation which leads to increase in PaCO_2_ and decrease in PO_2_. The incidence of hypoxia during ERCP with sedation has been reported to range from 16.2 to 39.2%^10, 11^, which is higher than those of other endoscopic procedures, presumably because ERCP procedures can be lengthy and are often performed in the prone position. The prone position increases the ventilator resistance of patients since thorax of patients is compressed against the exam bed and inducing upward displacement of abdominal viscera against the diaphragm difficult normal breathing^12^. Predictive factors of desaturation during endoscopy have been previously reported to be an age of > 60 years, an American Society of Anesthesiologists (ASA) class of > III^11^, BMI > 20 kg/m^2^, or the presence of a comorbidity such as hypertension (HTN), diabetes mellitus (DM), or heart disease^13^.

To prevent hypoxia during sedated endoscopy, close monitoring of airway, respiration, and oxygenation are critical. Humidified heated high-flow nasal oxygen (HFNO) delivered through a nasal cannula is a new type of oxygen therapy that provides oxygen containing heated, humidified air at a constant high flow rate. In addition, the high flow rates used create a “positive end expiratory pressure” (PEEP) that assist ventilation and reduce the work required for breathing^14, 15^. Furthermore, HFNO during sedation for flexible bronchoscopy has been shown to be safer in patients with stable respiratory parameters than Venturi masks and to provide better oxygenation^16^. The use of HFNO during procedural sedation has attracted interest, because it enables steady fractions of inspired oxygen (FIO_2_) at high airflow rates with minimal interference to endoscopic devices inserted through the oral route. To the best of our knowledge, recently no study nor case report has addressed the topic of HFNO during ERCP.

Therefore, we conducted this retrospective study to evaluate the effect of HFNO on desaturation events during ERCP sedation and to identify predictors of desaturation during ERCP.

## Materials and methods

### Participants

This study was conducted using a retrospective single-center design. A chart review was performed during the 4-month period from March 2019 to June 2019 of consecutive patients that underwent ERCP with HFNO under sedation. The inclusion criteria were; age > 19 years, level of consciousness (LOC) I which is alert or II which is drowsy, and the ability to cooperate with postural changes. The exclusion criteria were as follows: (1) a coagulation disorder or nasopharyngeal obstruction or bleeding tendency preventing nasal airway approach; (2) a tracheostomy or scheduled for airway intubation; (3) patients with home oxygen or respirator; (4) patients with a diagnosis of chronic destructive pulmonary disease (COPD), in whom high oxygen therapy might induce a narcotic condition; and (5) surgical failures caused by failed cannulation or altered anatomy. The patient characteristics investigated were age, gender, body mass index (BMI, kg/m^2^), and ASA physical status classification^17^, antiplatelet or anticoagulant use, and the presence of preexisting diseases such as HTN, DM, congestive heart failure (CHF), cerebral infarction, or myocardial infarction (MI) or coronary artery obstructive disease (CAOD). Data were analyzed after adjusting for sedative type such as midazolam or/and propofol, dose of sedative agent, procedure duration, and oxygen saturation (SpO_2_).

### Clinical care and equipment

**Figure 1:**
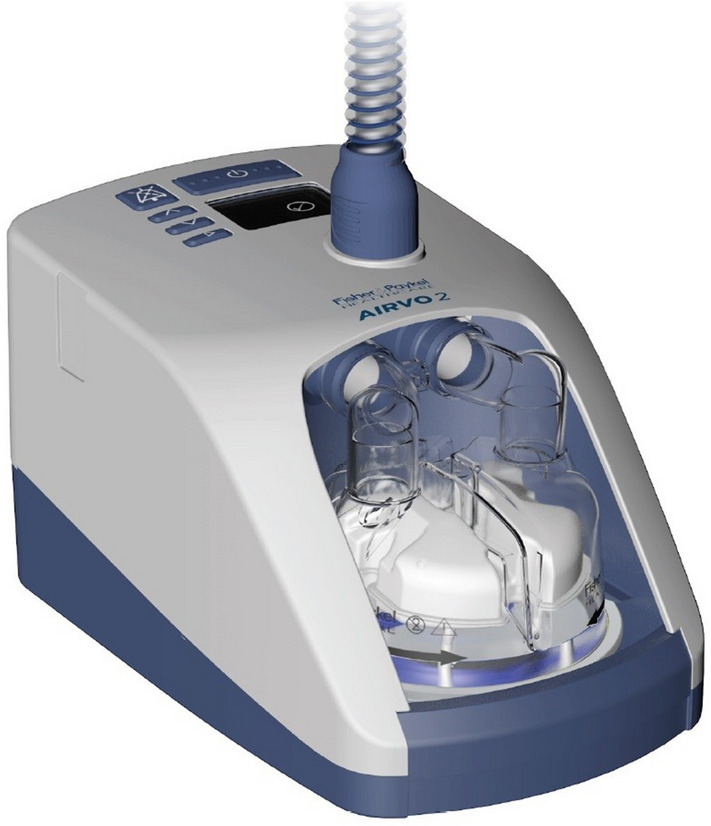
High flow nasal oxygen (Fisher and Paykel Healthcare Limited, Panmure, New Zealand).

Two of expert endoscopists performed all 262 ERCP procedures. Patients were positioned prone for ERCP and all were supplied HFNO by using the Optiflow oxygen delivery system (Fisher and Paykel Healthcare Limited, Panmure, New Zealand, Fig. 1). It delivers humidified oxygen via specifically designed high-flow nasal prongs that we modified for sedation care to capture end-tidal carbon dioxide (ETCO_2_). The HFNO settings were adjusted as follows; flow rate 50 L/min, FiO_2_ 50%, oxygen saturation ≥ 95%, and temperature and 37 °C. During endoscopy, the patient’s mouth is kept open because pressure is lower with an open mouth than a closed mouth and 1.7 cm H_2_O with an open mouth at a flow rate of 50 L/min, which is sufficient to maintain SpO_2_^18^. These settings are similar to those previously reported^19, 20^.

Sedation was achieved using midazolam and/or propofol; a propofol bolus was administered when a patient showed no sedative response to midazolam and/or propofol. For propofol, the initial bolus injection was 40 mg for patients < 70 years old, 30 mg for patients aged 70–89 years, and 20 mg for those aged ≥ 90 years. When the target sedation level was not obtained, additional injections of 20 mg propofol were given^21^. Maximum doses of propofol (3 mg/kg) and midazolam (10 mg) were limited, respectively. No opioids were used.

Standard monitoring, including heart rate, blood pressure, and SpO_2_ was performed (M20, MEDIANA, Korea). Total doses of propofol and midazolam were also recorded. When subclinical respiratory depression (90% ≤ SpO_2_ < 95%) occurred, it was corrected by opening the airway using the jaw-thrust maneuver. When peripheral arterial oxygen desaturation (SpO_2_ ≤ 90%) occurred, flow rate was firstly raised up to 60 L/min and if saturation doesn’t recover over 95%, then FIO_2_ was raised up to 100%. The upper limit of SpO_2_ and flow rate is 100% and 60 L/min. However, if desaturation was accompanied by agitation occurred in patients sedated using midazolam, flumazenil was directly administered. The interruption criteria for discontinuation of the endoscopic procedure were SpO_2_ ≤ 90% and no SpO_2_ recovery by HFNO.

### Measurement of outcomes

The main study outcome was the incidence of desaturation. Secondary outcomes were sedation-related adverse events, that is, agitation, tachycardia (heart rate > 100/min), or bradycardia (heart rate < 60/min).

### Statistical analysis

Categorical variables are presented as numbers (%) and numerical variables as means ± SDs or medians which defines minimum, maximum, or interquartile ranges. Numerical variables were analyzed using the independent-samples t-test, and categorical variables using Fisher’s exact test. Statistical significance was accepted for *p* values < 0.05, and the analysis was performed using SPSS Ver. 19.0 (SPSS Inc., Chicago, IL).

### Ethics

Written informed consent was obtained from all 262 study subjects prior to study commencement. All methods were carried out in accordance with relevant guidelines and regulations that was approved by the Institutional Review Board of Inha University Hospital (2019-06-038).

## Results

### Basic and clinical characteristics

Records of 262 ERCP cases performed in the endoscopy suite during the 4-month period from March 2019 to June 2019 at our institution were analyzed. Table 1 lists patients’ ages, genders, BMI, medical histories, and ASA classes. Mean age was 68.1 ± 16.5 years, 51.5% were male, and mean BMI was 23.9 ± 4.4 kg/m^2^. Among the patients’ medical histories including DM, HTN, CAOD, MI, CHF, and cerebral infarction, the largest number of patients had HTN (36.6%). 15.3% of patients were taking an anti-platelet or anti-thrombotic agent due to underlying diseases or as prophylaxis for vessel disease. ASA physical statuses were class I in 108 patients (41.2%), class II in 56 patients (21.4%), and class III in 98 patients (37.4%). Table 2 summarizes indications for ERCP; the most patients underwent ERCP for a common bile duct (CBD) stone (159 patients, 60.7%) and second most common was due to malignancies (61 patients, 23.3%) including cholangiocarcinoma (27 patients, 44.3%), gall bladder cancer (14 patients, 23.0%), pancreatic cancer (11 patients, 18.0%), ampulla of Vater (AoV) cancer (5 patients, 8.2%), and hepatocellular carcinoma (HCC) (4 patients, 6.6%). Minor causes of ERCP were intraductal papillary mucinous neoplasm (IPMN), choledochal cyst, sphincter of Oddi dysfunction (SOD), pancreatic pseudocyst, primary sclerosing cholangitis (PSC), pancreatic neuroendocrine tumor (NET), post cholecystectomy biliary leakage, and AoV adenoma.

**Table 1:** Basic characteristics of the patients that underwent ERCP under sedation.

Variables (n = 262)	
Age (year)	68.1 ± 16.0
**Gender**, n (%)	
Male	135 (51.5)
Female	127 (48.5)
BMI (kg/m^2^)	23.9 ± 4.4
**Comorbidity**	
HTN, n (%)	96 (36.6)
DM, n (%)	78 (29.7)
CAOD, MI, n (%)	16 (6.1)
CHF, n (%)	2 (0.8)
Cerebral infarction, n (%)	15 (5.7)
Antiplatelet, antithrombotic medication use (yes), n (%)	40 (15.3)
**ASA status**, n (%)	
I	108 (41.2)
II	56 (21.4)
III	98 (37.4)

ERCP, endoscopic retrograde cholangiography; BMI, body mass index; HTN, hypertension; DM, diabetes mellitus; CAOD, coronary artery obstructive disease; MI, myocardial infarction; CHF, congestive heart failure; ASA, the American Society of Anesthesiologists.

**Table 2:** Indication for ERCP.

CBD stone	159 (60.7)
**Malignancies**	61 (23.3)
Cholangiocarcinoma	27 (44.3)
Gall bladder cancer	14 (23.0)
Pancreatic cancer	11 (18.0)
AoV cancer	5 (8.2)
HCC	4 (6.6)
Benign structure	23 (8.8)
**Others**	19 (7.3)
SOD	5 (26.3)
Pancreatic pseudocyst	4 (21.1)
IPMN	3 (15.8)
Post cholecystectomy biliary leakage	3 (15.8)
Choledochal cyst	1 (5.3)
PSC	1 (5.3)
Pancreatic NET	1 (5.3)
AoV adenoma	1 (5.3)

ERCP, endoscopic retrograde cholangiography; CBD, common bile duct; AoV, ampulla of Vater; SOD, sphincter of Oddi dysfunction; IPMN, intrapapillary mucinous neoplasm; PSC, primary sclerosing cholangitis; NET, neuroendocrine tumor.

### Sedation protocol

Sedation type and sedative agent dose during ERCP were detailed in Table 3. Propofol was the most preferred sedative agent (172 patients, 65.6%) rather than midazolam (41 patients, 15.6%) or combined midazolam and propofol (49 patients, 17.8%). The mean doses of midazolam and propofol were 1.7 ± 2.5 mg and 105.9 ± 69.5 mg, respectively, which were below the maximum and safety dose^22, 23^. However, there was no significant difference in basic and clinical characteristics of patients depended on kinds of the sedative agents (Supplement Table 1). Mean procedural time for ERCP was 17.6 ± 10 min.

**Table 3:** Sedation protocol.

**Sedation type**	
Midazolam, n (%)	41 (15.6)
Propofol, n (%)	172 (65.6)
Midazolam plus Propofol	98 (37.4)
**Sedation dose**	
Midazolam (mg)	1.7 ± 2.5
Propofol (mg)	105.9 ± 69.5
**Procedure time (min)**	17.6 ± 10

### Clinical outcomes

The desaturation events occurred in 9 (3.4%) of the 262 patients during sedated ERCP (Table 4). Patients who experienced desaturation were significantly older than those that did not (desaturated vs. non-desaturated: 82.1 ± 9.6 vs. 67.6 ± 16, *p* < 0.05). Those with experienced desaturation received significantly higher doses of midazolam (5.6 ± 3.0 mg vs. 1.53 ± 2.4 mg, *p* < 0.05) or propofol (30 ± 79.4 mg vs. 108.6 ± 67.7 mg, *p* < 0.05). Furthermore, patients sedated with midazolam in the desaturation groups (7/9(77.8%)) had higher percentage than those sedated with propofol ((1/9(11.1%) *p* < 0.05)) or midazolam plus propofol ((1/9(11.1%), *p* < 0.05)). Among 172 patients sedated with propofol, only one patient experienced desaturation event. However, rates higher ASA classes ((class I: 3/9 (33.3%) vs. 105/253(41.5%), class II: 4/9 (44.4%) vs. 52/253(20.6%), class III: 2 (22.2%) vs. 96/253 (7.5%), *p* = 0.22), higher BMIs (21.8 ± 4.1 kg/m^2^ vs. 23.9 ± 0.5 kg/m^2^, *p* = 0.15), longer procedure times (15.2 ± 10.3 min vs. 17.7 ± 10 min, *p* = 0.39), overall comorbidities {HTN (5/9(55.6%) vs. 91/253(36%), *p* = 0.23), DM (1/9 (11.1%) vs. 77/253(30.4%), *p* = 0.21), CAOD or MI (0/9(0%) vs. 16/253(63%), *p* = 0.44), and CHF (0/9(0%) vs. 2/253 (0.8%), *p* = 0.48)} were not significantly related with desaturation.

**Table 4:** General characteristics of the desaturated and non-desaturated groups.

	Non-desaturated group (n = 253)	Desaturated group (n = 9)	*p* value
Age	67.6 ± 16	82.1 ± 9.6	< 0.05
Gender (male)	130 (51.4)	5 (55.6)	0.81
**Comorbidities**			
BMI	23.9 ± 0.5	21.8 ± 4.1	0.15
HTN	91 (36)	5 (55.6)	0.23
DM	77 (30.4)	1 (11.1)	0.21
CAOD, MI	16 (63.0)	0 (0)	0.44
CHF	2 (0.8)	0 (0)	0.48
Cerebral infarction	14 (5.5)	1 (11.1)	0.48
Antiplatelet or anticoagulant	39 (51.4)	1 (11.1)	0.72
**ASA class**			0.22
I	105 (41.5)	3 (33.3)	
II	52 (20.6)	4 (44.4)	
III	96 (7.5)	2 (22.2)	
Procedure time (min)	17.7 ± 10	15.2 ± 10.3	0.39
**Sedative**			< 0.05
Midazolam	34 (13.4)	7 (77.8)	
Propofol	171 (67.6)	1 (11.1)	
Midazolam plus Propofol	48 (19)	1 (11.1)	
**Dose of sedative agent (mg)**			
Midazolam	1.53 ± 2.4	5.6 ± 3.0	< 0.05
Propofol	108.6 ± 67.8	30 ± 79.4	< 0.05

BMI, body mass index; HTN, hypertension; DM, diabetes mellitus; CAOD, coronary artery obstructive disease; MI, myocardial infarction; CHF, congestive heart failure; ASA, the American Society of Anesthesiologists.

Anthropomorphic and clinical characteristics of nine desaturated patients are summarized in Table 5. All underwent ERCP because of a CBD stone. Seven of the nine showed agitation (7/9, 78%), one tachycardia (heart rate 120/min), and one bradycardia (heart rate 50/min) during desaturation. None of the desaturated patients was intubated. However, the procedure was stopped in one patient because of delayed recovery from awakening.

**Table 5:** Basic and clinical characteristics of desaturated patients.

Age	Gender	BMI (kg/m^2^)	ASA class	Indication for ERCP	Procedure time (min)	Type and dose of sedative	Accompanying symptom	Treatment
84	Female	24.9	III	CBD Stone	6	Midazolam (5 mg)	Agitation	Flumazenil
84	Male	21.1	I	CBD Stone	32	Midazolam (10 mg)	Agitation	Flumazenil
88	Female	22.6	II	CBD Stone	6	Midazolam (10 mg)	Bradycardia	Flumazenil
92	Female	18.2	I	CBD Stone	27	Midazolam (5 mg)	Tachycardia	Flumazenil HFNO 0.7/50
89	Male	28.4	II	CBD Stone	25	Midazolam (5 mg)	Agitation	Flumazenil HFNO 0.6/50
79	Female	16.9	II	CBD Stone	12	Midazolam plus Propofol (5 mg plus 30 mg)	Agitation	Flumazenil HFNO 0.7/50
59	Male	26.6	II	CBD Stone	13	Propofol (240 mg)	Agitation	HFNO 0.7/50, Stop ERCP
82	Male	18.7	I	CBD Stone	13	Midazolam (5 mg)	Agitation	Flumazenil HFNO 0.7/50
82	Male	18.5	III	CBD Stone	3	Midazolam (5 mg)	Agitation	Flumazenil HFNO 0.7/50

BMI, body mass index; HTN, hypertension; DM, diabetes mellitus; CAOD, coronary artery obstructive disease; MI, myocardial infarction; CHF, congestive heart failure; ASA, the American Society of Anesthesiologists; CBD, common bile duct; HFNO, high flow nasal oxygen.

## Discussion

In this study, we investigated the effect of HFNO on desaturation events during sedated ERCP and attempted to identify predictors of desaturation. Of the 262 patients administered HFNO during sedated ERCP, only 9 patients (3.8%) experienced desaturation. Desaturation rates during ERCP under sedation have been reported to range from 16.2 to 39.2%^10, 11^. As compared to the reference range of desaturation rate during ERCP with conventional low-flow oxygen under sedation, the present study shows ERCP with high-flow nasal oxygen under sedation dramatically reduced desaturation rates during ERCP.

Lee et al. (2018) first reported on a trial of HFNO during endoscopy with respect to its preventive effect against hypoxia in obese patients receiving colonoscopy^24^. However, no significant difference (*p* = 0.79) was observed between the desaturation rates of patients that received HFNO (11/28, 39.3%) or a nasal cannula (14/31, 45.2%). However, in a recent prospective, multicenter study, Lin et al.^19^ reported a desaturation rate of only 1.6% during upper endoscopy under HFNO sedation, which is much lower than the desaturation rate observed in the present study. We attribute this difference to the characteristics of ERCP, which is more advanced procedure with a longer procedure time in the prone position.

Many studies have been conducted to identify predictive factors of desaturation during sedated endoscopy in the context of preventing hypoxic events. An age > 60 years and an ASA class of > III have been suggested to be candidate factors during ERCP^11^, and a BMI > 20 kg/m^2^, the presence of comorbidities such as DM, HTN, cardiovascular disease, and combined upper and lower endoscopy rather than single endoscopy have been reported to be risk factors of desaturation during endoscopy^13^. In the present study, age, sedation dose, and the use of midazolam were found to be significantly associated with desaturation during ERCP under sedation (Table 4). However, somewhat unexpectedly ASA class (*p* = 0.22), BMI (*p* = 0.15), procedure time (*p* = 0.39), and the presence of a comorbidity (e.g., HTN, DM, or heart disease) were not significantly associated with desaturation. This may have been due to the low number of desaturated patients (9 of 262), which would have limited statistical power. Interestingly, we also found the use of midazolam was significantly higher in patients that experienced desaturation. It has been previously reported that the incidence of hypoxia during upper endoscopy in liver cirrhosis patients was greater for midazolam than propofol^25^, which indicates propofol is safer during endoscopy.

Of symptoms that accompanied desaturation, agitation was the most common for midazolam and propofol. However, it is not clear whether agitation was the result of desaturation or of sedation-related adverse events, because in a large prospective multicenter study, only 9 (0.19%) of 314,190 patients who underwent sedated endoscopy showed agitation without any desaturation events^26^.

ERCP procedures were completed in 8 of the 9 desaturation cases. In the other patient, the procedure was stopped because of delayed recovery from awakening. The dose of propofol administered to this patient was relatively high which was 240 mg that is ~ 3 mg/kg. Despite the many advantages of propofol such as its ultra-short onset and short recovery time^27, 28^, it has well-known disadvantages including its dose-dependent abilities to induce general anesthesia or hemodynamic and respiratory depression, and the lack of a pharmacologic antagonist^29, 30^. This event was compatible with our data in which even though propofol was less used in desaturated group, still higher dose of propofol was significantly related with desaturation. Furthermore, recent studies have recommended propofol and midazolam be used in combination rather than as single agents during sedated endoscopy because synergy between the two has a dose-saving effect^31, 32^. Thus, as a higher single dose of propofol might delay awakening the combined use of propofol and midazolam might be more suitable.

The limitations of the present study are as follows. First, it is limited by its retrospective design the lack of a control group. Furthermore, sedation scale or clinically important outcomes (e.g., the incidence of post-procedural abdominal distention and pain) were not available from medical records. Second, we used SpO_2_ to identify desaturation, and did not use other parameters, such as partial pressure of oxygen (PO_2_). However, pulse oximetry is a standard for monitoring patients during sedation and provides a noninvasive, sensitive means of monitoring peripheral oxygen saturation. Third, we did not measure ETCO_2_ to detect subclinical respiratory depression, such as hypercapnia or apneic episodes, though we did observe chest movements closely so as not to miss apneic episodes. Fourth, we excluded patients diagnosed with COPD based on the consideration that high oxygen therapy might induce a narcotic condition. However, we believed that HFNO can protect patients from exposure to narcotic condition even in high FIO_2_ since HFNO is able to provide increasing end-expiratory lung volume of patients through maintaining positive airway pressure to airway. We suggest patients with higher ASA classes and advanced lung disease patients be investigated to confirm the ability of HFNO to reduce hypercapnia^33^.

In conclusion, our study shows HFNO supportive oxygen therapy can prevent desaturation in patients undergoing sedative ERCP and that older age, sedation dose, and the use of midazolam predict desaturation during ERCP. However, we believed that large-scale, randomized, and comparative studies would be required to confirm our results.

## Supplementary Information

Supplementary Tables

## Abbreviations

GI: Gastrointestinal
ERCP: Endoscopic retrograde cholangiography
ASA: American Society of Anesthesiologists
HTN: Hypertension
DM: Diabetes mellitus
HFNO: High-flow nasal oxygen
PEEP: Positive end expiratory pressure
FIO_2_: Fractions of inspired oxygen
LOC: Level of consciousness
COPD: Chronic destructive pulmonary disease
BMI: Body mass index
CHF: Congestive heart failure
MI: Myocardial infarction
CAOD: Coronary artery obstructive disease
SpO_2_: Oxygen saturation
ETCO_2_: End-tidal carbon dioxide
CBD: Common bile duct
AoV: Ampulla of Vater
IPMN: Intraductal papillary mucinous neoplasm
SOD: Sphincter of Oddi dysfunction
PSC: Primary sclerosing cholangitis
NET: Neuroendocrine tumor
