# Supplementary material for: Clinical efficacy of high-flow nasal oxygen in patients undergoing ERCP under sedation
Source: Sci Rep. 2021 Jan 11;11:350. doi: 10.1038/s41598-020-79798-7 (PMC7801411; doi:10.1038/s41598-020-79798-7)
Supplement: Supplementary file 1 — Supplementary Tables [file 41598_2020_79798_MOESM1_ESM.docx]

**Clinical efficacy of high-flow nasal oxygen in patients undergoing ERCP under sedation**

^§1^Boram Cha, ^§2^Man-Jong Lee, *^1^Jin-Seok Park, ^1^Seok Jeong, ^1^Don Haeng Lee, ^1^Tae Gyu Park

**Supplement Table 1. Basic and clinical characteristics among midazolam, propofol, or Midazolam plus Propofol used groups**

|  | **Midazolam**  **(n=41)** | **Propofol**  **(n=172)** | **Midazolam plus Propofol**  **(n=49)** | **p-value** |
| --- | --- | --- | --- | --- |
| **Gender** (male), n(%) | 14(34.1) | 90(52.3) | 31(63.3) | 0.02 |
| **Age** (year) | 82±8 | 65±14 | 66±19 | 0.92 |
| **BMI** (kg/m^2^) | 22.4±4.5 | 24.2±4.3 | 24.2±4.5 | 0.06 |
| **Antiplatelet use** (yes), n(%)  **Comorbidity** | 12(29.3) | 24(14) | 4(8.2) | 0.16 |
| DM, n(%) | 8(19.5) | 52(30.2) | 18(36.7) | 0.20 |
| HTN, n(%) | 19(46.3) | 57(33.1) | 20(40.8) | 0.23 |
| CAOD, MI, n(%) | 3(7.3) | 11(6.4) | 2(4.1) | 0.79 |
| CHF, n(%) | 0 | 2(1.2) | 0 | 0.59 |
| Cerebral infarct, n(%) | 6(14.6) | 7(4.1) | 2(4.1) | 0.03 |
| **ASA status**, n(%)  I  II  III | 15(36.6)  10(24.4)  16(39) | 75(43.6)  35(20.3)  62(36) | 18(36.7)  11(22.4)  20(40.8) | 0.63 |
| **Desaturation event**  (yes), n(%) | 7(17.1) | 1(0.6) | 1(2) | <0.01 |
| **Procedure time** (min) | 16±9 | 16 | 20±13 | 0.10 |

Abbreviations: BMI; body mass index, DM; diabetes mellitus, HTN; hypertension, CAOD; coronary artery obstructive disease, MI; myocardial infarction, CHF; congestive heart failure, ASA; the American Society of Anesthesiologists
